# Anxiety- and depression-related somatic symptoms and atlantoaxial disorders: potential mechanistic links and clinical implications

**DOI:** 10.3389/fpsyt.2026.1919035

**Published:** 2026-09-03

**Authors:** Shuxian Yang, Qianru Li, Wanying Shi, Xiaoyun Liu

**Affiliations:** 1 The First Hospital of Hebei Medical University (Hebei Hospital of Xuanwu Hospital, Capital Medical University), Shijiazhuang, Hebei, China; 2 Plastic Surgery Hospital, Chinese Academy of Medical Sciences and Peking Union Medical College, Beijing, China; 3 Shijiazhuang People's Hospital / Shijiazhuang First Hospital, Shijiazhuang, Hebei, China

**Keywords:** anxiety, atlantoaxial joint, depression, psychosomatics, somatic symptom disorder

## Abstract

**Background:**

Anxiety and depressive disorders often include pain, dizziness, headache, fatigue, nausea, and sleep disturbance. These symptoms may also occur in atlantoaxial disorders, but overlap does not establish a shared cause.

**Objective:**

To examine whether clinical or mechanistic evidence supports an association between objectively defined atlantoaxial disorders and somatic symptom burden in people with anxiety or depression, while distinguishing direct evidence from inferences based on general neck-pain research.

**Methods:**

PubMed/MEDLINE, Embase, Scopus, Web of Science Core Collection, and APA PsycInfo were searched from inception to 29 July 2026. Two complementary searches were conducted: the first combined atlantoaxial/C1–C2 conditions with anxiety, depression, or somatic-symptom concepts, and the second combined adjacent cervical conditions with the same psychiatric and somatic-symptom concepts. Evidence was classified as direct or partial-direct, adjacent, mechanistic inference, or hypothesis-generating. Because populations, diagnoses, and outcomes were heterogeneous, findings were synthesized narratively rather than pooled.

**Results:**

The searches retrieved 8,882 records, of which 5,225 remained after deduplication. Only three studies provided partial-direct evidence by including both an upper-cervical variable and a psychiatric variable in the same population; two used imaging-based spinal assessments, whereas one relied on administrative diagnosis and procedure codes. None was designed to investigate somatic symptom disorder, none established that anxiety or depression caused structural C1–C2 disease, and none demonstrated a bidirectional causal relationship. General neck-pain studies support associations between psychological distress and pain-related outcomes, while studies of cervical dizziness and proprioception provide possible adjacent mechanisms. Autonomic, central pain-processing, inflammatory, sleep-related, and biomechanical pathways remain predominantly indirect or hypothetical in relation to atlantoaxial disease.

**Conclusions:**

Atlantoaxial disorders and anxiety- or depression-related somatic symptoms may coexist and may have overlapping clinical presentations. Current evidence is insufficient to quantify a specific association or infer causality. Clinical assessment should prioritize neurological and vascular red flags, objective definition of C1–C2 pathology, clinicoradiological concordance, exclusion of common vestibular and neurological causes of dizziness, and parallel assessment of anxiety, depression, sleep disturbance, fear avoidance, and somatic symptom burden. Prospective studies using standardized imaging criteria and validated psychiatric measures are needed.

## 1 Introduction

Anxiety and depressive disorders are major contributors to global disability (1). Their clinical expression frequently includes bodily symptoms, including pain, dizziness, headache, fatigue, gastrointestinal discomfort, palpitations, and sleep disturbance. These symptoms may represent manifestations of an affective disorder, adverse effects of treatment, a coexisting medical condition, heightened symptom monitoring, or a separate diagnosis such as somatic symptom disorder (SSD). They should not be treated as interchangeable constructs.

Atlantoaxial disorders are a heterogeneous group of structural or dynamic conditions involving the atlas (C1), axis (C2), their articulations, and stabilizing ligaments. Depending on cause and severity, they range from incidental degenerative change to traumatic or inflammatory instability with spinal-cord or vertebral-artery risk. Their most defensible clinical manifestations are upper-neck or occipital pain, restricted motion, torticollis in rotatory disorders, and neurological deficits when neural compression is present. Dizziness, nausea, tinnitus, and visual complaints have been described, but their frequency and attribution to C1–C2 pathology are uncertain.

In general cervical-pain populations, psychological distress, sleep disturbance, and other psychosocial factors have been associated with pain, disability, and related health outcomes (2, 3). These findings are clinically important, but they cannot automatically be extrapolated to objectively confirmed atlantoaxial instability, subluxation, rotatory fixation, or degeneration.

The central review question was therefore: Does available clinical or mechanistic evidence support an association between objectively defined atlantoaxial disorders and somatic symptom burden in patients with anxiety or depression? We additionally examined how clinicians might evaluate overlapping symptoms without reducing them to either a purely structural or purely psychological explanation.

## 2 Methods

### 2.1 Review design and scope

This structured narrative review was prepared with reference to the Scale for the Assessment of Narrative Review Articles (SANRA) (4). A meta-analysis was not planned because the literature did not provide a sufficiently homogeneous set of populations, atlantoaxial definitions, psychiatric constructs, comparators, or outcomes. The review focused primarily on adults. Pediatric or adolescent studies were included only to clarify rotatory fixation or developmental instability, or as explicitly indirect contextual evidence when relevant adult evidence was limited.

The primary clinical phenomenon was somatic symptom burden associated with anxiety or depressive disorders, not SSD alone. SSD was addressed as a distinct diagnostic construct because it is frequently confused with having multiple physical symptoms. The exposure of interest was an objectively defined atlantoaxial disorder, including instability, subluxation, rotatory fixation/subluxation, inflammatory disease, traumatic disease, or degenerative C1–C2 pathology. Non-specific neck pain and clinically defined upper-cervical dysfunction were treated as adjacent rather than equivalent conditions.

### 2.2 Information sources and search strategy

PubMed/MEDLINE, Embase, Scopus, Web of Science Core Collection, and APA PsycInfo were searched from database inception to 29 July 2026. No language, publication-date, or study-design filters were applied at the search stage. Conference records, trial registrations, case reports, reviews, and non-human studies were retained during initial retrieval so that their evidentiary role could be evaluated explicitly.

Two complementary search blocks were used. The direct search combined terms for atlantoaxial/C1–C2 conditions (for example, *atlantoaxial*, *atlanto-axial*, *C1-C2*, *atlas-axis*, and *upper cervical instability*) with terms for anxiety, depression, somatic symptoms, SSD, somatization, psychosomatic symptoms, persistent physical symptoms, bodily distress, health anxiety, symptom amplification, and psychological distress. The adjacent search combined terms for neck pain, cervical pain, upper-cervical dysfunction, cervicogenic dizziness, cervical dizziness/vertigo, and cervicogenic headache with the same psychiatric and somatic-symptom block. Complete database-specific strategies are provided in **Supplementary Appendix 1**.

### 2.3 Eligibility and selection

Evidence was eligible for the primary synthesis when it met one of four prespecified categories:

1. Direct or partial-direct evidence: psychiatric or somatic-symptom data and an upper-cervical assessment were available in the same participants. Evidence was classified as direct when objectively defined C1–C2 pathology and a targeted psychiatric or somatic-symptom measure were analyzed in relation to one another. Evidence was classified as partial-direct when that relationship was not directly tested, or when a spinal or psychiatric variable relied on administrative coding or a non-targeted assessment.
2. Adjacent evidence: mental-health variables were examined in general neck pain, cervical radiculopathy, whiplash-associated disorders, cervicogenic headache, or cervical-dizziness populations without a specific C1–C2 diagnosis.
3. Mechanistic inference: autonomic, inflammatory, interoceptive, nociceptive, proprioceptive, sleep, or behavioral findings were reported without atlantoaxial assessment.
4. Hypothesis-generating evidence: a biologically plausible pathway or small uncontrolled clinical observation had not been validated in a comparative human study.

Records were excluded from the evidentiary synthesis when the terms were used in unrelated contexts; when atlantoaxial disease and psychiatric symptoms were merely listed as separate comorbidities; when a case report was used to imply symptom frequency, diagnostic accuracy, or treatment efficacy; or when general neck-pain evidence was presented as proof of structural C1–C2 disease. Foundational anatomy, diagnostic guidance, consensus statements, and measurement studies were retained as contextual references.

Titles and abstracts were screened in a single-reviewer workflow using predefined criteria. Potentially relevant direct records and key adjacent studies underwent full-record assessment. Because screening was performed by one reviewer, an inter-reviewer disagreement-resolution procedure was not applicable; the resulting risk of selection and interpretation bias is acknowledged in the Limitations. Citation lists of included reviews and consensus statements were also checked for foundational sources.

### 2.4 Data extraction and appraisal

For clinically relevant studies, we extracted design, sample, spinal diagnosis and assessment method, psychiatric or somatic-symptom measure, principal finding, and limitations. The primary appraisal domains were study design, diagnostic validity, directness to C1–C2 disease, psychiatric measurement quality, control of confounding, and precision. Study selection and citation were guided by clinical directness, diagnostic validity, methodological relevance, and evidentiary role rather than publication year. Recent studies were not preferentially included solely because of recency; foundational diagnostic, consensus, measurement, and mechanistic sources were retained when they remained authoritative. A single formal risk-of-bias instrument was not applied across the entire evidence base because it included diagnostic reviews, consensus statements, cross-sectional studies, administrative cohorts, mechanistic studies, and case reports. This is a limitation of the review. Certainty language was calibrated to study design and directness; case reports and uncontrolled series were treated as hypothesis-generating only.

## 3 Search results and evidence landscape

The direct searches retrieved 445 records and the adjacent searches retrieved 8,437 records, for a total of 8,882 records. After removal of 3,657 duplicates, 5,225 unique records remained: 315 from the direct-search stratum and 4,910 from the adjacent-only stratum.

The direct stratum contained many false-positive or clinically non-specific co-mentions. Common examples included Down syndrome reviews that separately discussed depression and atlantoaxial screening, rheumatoid-arthritis reviews listing depression and cervical disease as different comorbidities, surgical studies recording depression only as an administrative variable, and papers in which “C1–C2” or “axis” referred to an unrelated experimental coordinate. Three studies provided partial-direct evidence, but none directly investigated SSD or anxiety/depression-related somatic symptom burden as a function of objectively defined C1–C2 pathology (5–7).

No study was found that recruited patients with anxiety, depression, or SSD and systematically evaluated them for atlantoaxial disease. No prospective study established that psychiatric symptoms preceded structural C1–C2 change. No controlled study demonstrated a bidirectional causal relationship. The most relevant evidence is summarized in **Table 1**.

**Table 1:** Evidence most relevant to the proposed relationship.

Evidence level	Study and design	Spinal assessment	Psychiatric/somatic assessment	Main contribution	Principal limitation
Partial-direct	Suzuki et al., 2023; cross-sectional rheumatoid-arthritis cohort, n=146 (5)	Plain radiography and MRI; upper-cervical lesions and alignment	Questionnaire assessment of anxiety, depression, quality of life, and neck pain	Upper-cervical lesions were associated with neck pain	The study was not designed to test an upper-cervical lesion–psychiatric association; SSD was not assessed
Partial-direct	Ulutatar et al., 2019; rheumatoid-arthritis cohort plus matched controls, n=212 (106 patients with rheumatoid arthritis and 106 matched controls) (6)	Cervical radiography for subluxation; cervical joint-position error	Depression, fatigue, quality of life, and balance scales	Atlantoaxial subluxation was associated with impaired proprioception; depression showed weak or non-significant relationships with proprioception	Cross-sectional; inflammatory systemic disease; no specific test of AAS versus depression
Partial-direct	Tanenbaum et al., 2016; administrative cohort of 8,189 adult atlantoaxial-fusion admissions (7)	Procedure and diagnosis codes	Depression coded as a comorbidity	Depression was one of several administratively coded comorbidities associated with selected inpatient outcomes	Administrative coding, no validated symptom assessment, no untreated comparator, and no evidence about disease causation
Hypothesis-generating	Goel et al., 2024; uncontrolled post-traumatic case series, n=14 (38)	Static and dynamic imaging using a nonstandard “central atlantoaxial dislocation” construct	Antidepressant use noted; no validated psychiatric assessment	Reports coexisting disabling symptoms and medication use before fixation	No control group, subjective outcomes, disputed diagnostic construct, selection bias, and inability to infer efficacy or causation
Adjacent	Mansfield et al., 2023; systematic review of cervical pain (2)	General cervical pain with/without radiculopathy	Psychosocial and mental-health variables	Psychological factors may be associated with pain, disability, and health outcomes	Substantial heterogeneity and low/very-low certainty; not specific to C1–C2
Adjacent	Liu et al., 2018; systematic review/meta-analysis (33)	Neck pain, not atlantoaxial disease	Anxiety/depression symptoms	Supports an association between neck pain and affective symptoms	Predominantly observational evidence; geographic and methodological heterogeneity
Adjacent	Seemungal et al., 2022; Bárány Society position (13)	Cervical dizziness literature	Differential diagnosis includes psychological causes	Emphasizes diagnostic uncertainty and absence of an accepted clinical test for cervical dizziness	Position statement; does not establish a C1–C2 mechanism
Adjacent	De Vestel et al., 2022; systematic review/meta-analysis (14)	Cervicogenic-dizziness populations	Dizziness and related outcomes	Some rehabilitation interventions may reduce symptoms in selected patients	Diagnostic heterogeneity; evidence does not concern structural atlantoaxial instability
Mechanistic inference	Li et al., 2022; narrative review (35)	Cervical proprioception and dizziness	Not a psychiatric cohort	Provides a plausible sensory-mismatch model	Narrative and cervical-general; not proof of atlantoaxial causation
Mechanistic inference	Yu et al., 2023; neuroimaging study (34)	No cervical assessment	Somatic symptoms in anxiety/depression	Reports altered resting-state functional connectivity associated with somatic symptoms	Cannot establish a peripheral cervical source
Mechanistic inference	Park et al., 2024; SSD neuroimmune study (36)	No cervical assessment	SSD symptoms and affective dysfunction	Reports associations among neuroimmune markers, functional connectivity, affective dysfunction, and somatic symptoms	Does not demonstrate local C1–C2 inflammation or instability
Mechanistic inference	Andias and Silva, 2020; systematic review (3)	Adolescent neck pain	Sleep and psychosocial variables	Supports sleep/psychosocial associations with neck pain	Age group and non-specific neck-pain population limit directness

## 4 Atlantoaxial disorders: definitions, diagnosis, and management

### 4.1 Anatomy and operational terminology

The atlantoaxial complex contains the median atlanto-odontoid articulation, paired lateral joints, joint capsules, the transverse ligament, alar ligaments, apical ligament, and other craniovertebral stabilizers. C1–C2 contributes approximately half of cervical axial rotation while maintaining proximity to the spinal cord, lower brainstem, C2 nerve root, and vertebral arteries (8, 9). High mobility does not itself imply pathological instability.

Several partly overlapping terms require separation. **Table 2** summarizes the principal entities, preferred imaging approaches, and major warning signs:

**Table 2:** Clinically distinct atlantoaxial entities.

Entity	Operational definition	Common causes	Typical or defensible presentation	Preferred imaging	Major red flags
Traumatic AAI/dislocation	Abnormal translation or rotation after trauma with ligamentous or bony injury	High-energy trauma, odontoid/atlas fracture, transverse-ligament injury	Severe upper-neck pain, restricted motion; possible neurological deficit	CT for bone/alignment; MRI for ligament, cord, and soft tissue	Weakness, sensory level, gait change, respiratory compromise, severe trauma
Inflammatory AAS	C1–C2 malalignment related to inflammatory synovitis and ligament/bone damage	Rheumatoid arthritis and other inflammatory arthropathies	Neck/occipital pain; may be silent until neurological compromise	Lateral radiographs; specialist-guided dynamic radiographs only when clinically appropriate and safe; MRI for pannus/cord; CT for bone	Myelopathy, progressive pain, long-tract signs, reduced posterior canal space
Developmental/congenital instability	Abnormal C1–C2 anatomy or laxity associated with congenital conditions	Os odontoideum, skeletal dysplasia, Down syndrome, congenital anomalies	Variable; may be asymptomatic or present with pain/myelopathy	Radiographs plus CT/MRI according to the suspected lesion	New neurological symptoms, bowel/bladder change, gait deterioration
AARF/AARS	Fixed or pathological rotatory relationship of C1–C2	Trauma, infection/inflammation, congenital predisposition	Painful torticollis and markedly restricted rotation	CT is usually most informative; MRI if ligament/cord concerns	Neurological signs, trauma, systemic infection, progressive deformity
Degenerative C1–C2 arthropathy	Structural degenerative change of the median or lateral joints	Age-related degeneration; prior inflammation or trauma	Predominantly upper-neck/occipital pain and restricted rotation when symptomatic	CT for osseous change; MRI for neural/soft-tissue questions	Myelopathy, severe unremitting pain, systemic disease
Non-specific upper-cervical dysfunction	Clinical pain or movement impairment without objective instability	Multifactorial musculoskeletal pain	Mechanical neck pain, restricted motion, headache in selected patients	Imaging usually not required without red flags	Any neurological/vascular warning sign or major trauma

Definitions, imaging approaches, and warning signs were synthesized from references (8–12, 15).

- Atlantoaxial instability (AAI) refers to pathological excessive translation or rotation between C1 and C2 caused by ligamentous, osseous, congenital, inflammatory, degenerative, or traumatic failure. It is a dynamic concept and should be supported by appropriate imaging and clinical context.
- Atlantoaxial subluxation (AAS) describes abnormal C1–C2 alignment on imaging. It can be anterior, posterior, lateral, vertical, or rotatory. A radiographic subluxation does not automatically establish dynamic instability or symptom causation.
- Atlantoaxial rotatory fixation/rotatory subluxation (AARF/AARS) describes a pathological, often fixed rotational relationship of C1 relative to C2, commonly presenting with painful torticollis and restricted rotation. Trauma and upper-respiratory inflammation are important causes, particularly in children (9).
- Degenerative atlantoaxial disease refers to C1–C2 joint-space loss, osteophytes, subchondral sclerosis/cysts, or related degenerative change. Imaging severity and clinical significance must be distinguished.
- Upper-cervical dysfunction is a clinical description of pain, restricted movement, or sensorimotor impairment involving the upper cervical region without demonstrated structural instability. It is not synonymous with AAI or AAS.

### 4.2 Imaging interpretation and diagnostic uncertainty

Plain radiography, CT, and MRI provide different information. On a neutral lateral radiograph, an anterior atlantodental interval greater than 3 mm in an adult is generally abnormal, but a single threshold does not define the cause, chronicity, dynamic behavior, or clinical importance of the finding (8, 10). The posterior atlantodental interval, or space available for the cord, is clinically important; values below approximately 14 mm have been associated with increased concern for cord compromise in selected populations (8, 10). These thresholds should support, rather than replace, etiological and neurological assessment.

Open-mouth odontoid radiographs can demonstrate gross malalignment, but lateral-mass asymmetry is sensitive to head rotation and beam orientation. Apparent asymmetry alone has led to unnecessary investigation and should not be treated as proof of AAI (11). CT is preferable for evaluating fractures, osseous morphology, articular congruity, and complex rotational abnormalities (8, 10, 12). MRI is indicated when ligament injury, inflammatory pannus or synovitis, spinal-cord compression or signal change, neural compromise, infection, or other soft-tissue disease is suspected (8, 10). Dynamic imaging may be useful in selected cases, but techniques and diagnostic thresholds vary, and movement should not be provoked when acute instability or neurological risk is suspected (10).

Radiological variation must be separated from clinically significant instability. A causal attribution is strongest when a plausible lesion, concordant symptoms/signs, and an appropriate temporal pattern occur together. Mild degeneration, asymmetry, or anatomical variation is common enough that an incidental finding must always be considered.

### 4.3 Clinical manifestations and cervical dizziness

Across heterogeneous etiologies, upper-neck or occipital pain, restricted rotation, stiffness, and torticollis in rotatory disorders are the most consistent local manifestations (8, 9). Spinal-cord compression can produce hand clumsiness, weakness, sensory disturbance, hyperreflexia, imbalance, or gait deterioration. Vertebral-artery compromise is uncommon but potentially serious and requires vascular assessment when symptoms and circumstances are compatible.

Potential vertebral-artery involvement is most relevant after upper-cervical trauma involving a C1 ring (Jefferson) fracture or odontoid injury, marked atlantoaxial displacement or rotatory dislocation, and in congenital or deformity states and anatomical variants that distort the artery; an additional iatrogenic risk arises during C1–C2 instrumentation (8, 10, 12). Stable degenerative arthropathy or non-specific upper-cervical dysfunction should not be presumed to cause vascular compromise solely because dizziness is present. CT angiography or MR angiography should be selected according to the trauma mechanism, vascular symptoms or signs, planned instrumentation, and local protocols rather than used routinely.

The literature does not permit reliable estimates of the frequency of dizziness, tinnitus, nausea, visual symptoms, or diffuse autonomic complaints specifically attributable to atlantoaxial disease. Such symptoms should therefore not be described as common manifestations without disease-specific cohort data. The Bárány Society concluded that evidence for a mechanistic link between neck pathology and true spinning vertigo is limited and that no accepted clinical diagnostic test establishes “cervical dizziness” (13). Before a cervical explanation is adopted, clinicians should consider vestibular migraine, benign paroxysmal positional vertigo, persistent postural-perceptual dizziness, central neurological disease, vascular disease, visual disorders, medication effects, orthostatic syndromes, panic/anxiety, and other relevant causes.

### 4.4 Management according to diagnostic category

Management differs fundamentally among non-specific neck pain, stable degeneration, confirmed instability, and neurological or vascular emergencies.

For non-specific neck pain or carefully diagnosed cervical dizziness without instability, education, graded exercise, sensorimotor rehabilitation, and selected mobilization/manual approaches may be reasonable (14, 15). This evidence cannot be generalized to structural AAI. For stable degenerative C1–C2 disease, treatment may include activity modification, analgesic strategies, and supervised rehabilitation, with specialist assessment for persistent severe symptoms or diagnostic uncertainty.

For confirmed atlantoaxial instability or dislocation, management is etiology- and severity-specific and should involve an appropriate spine specialist. High-velocity cervical manipulation should not be performed when atlantoaxial instability, fracture, dislocation, destructive inflammatory disease, acute myelopathy, ligamentous failure, or significant vascular risk is present or suspected (15, 16). Traction or forceful cervical movement should not be described as routine treatment for structurally unstable disease; the available evidence does not establish its general safety or efficacy.

Urgent referral is required for progressive neurological deficit, myelopathic signs, suspected acute fracture or ligament failure, severe trauma, spinal-cord compression, infection, or symptoms suggesting vertebrobasilar ischemia. Surgical stabilization is considered when there is clinically significant instability, irreducible deformity, progressive neurological compromise, cord compression, or failure of appropriate non-operative management (8).

## 5 Somatic symptoms, SSD, and differential diagnosis

### 5.1 Somatic symptom burden is not equivalent to SSD

Somatic symptoms are bodily experiences reported by a patient; they occur in medical illness, psychiatric illness, medication effects, functional disorders, and healthy populations. Anxiety and depression can include prominent somatic symptoms without meeting criteria for SSD. Conversely, SSD can coexist with a documented structural disorder, including a spinal disorder.

Under DSM-5-TR, SSD requires one or more distressing somatic symptoms together with disproportionate and persistent thoughts, anxiety, or behaviors related to the symptoms (17, 18). Symptom number, anatomical distribution, or absence of a medical explanation is not sufficient for diagnosis. The diagnosis should not be inferred merely because investigations are normal or because anxiety or depression is present.

ICD-11 uses the diagnosis of bodily distress disorder, which overlaps substantially with, but is not identical to, DSM-5-TR somatic symptom disorder. Both frameworks emphasize distressing bodily symptoms and excessive symptom-related attention or preoccupation, although their terminology, diagnostic wording, thresholds, and broader classification contexts differ (17, 18). Reviews should therefore specify the diagnostic framework used rather than treat somatic symptom disorder, bodily distress disorder, functional somatic symptoms, and persistent physical symptoms as interchangeable terms.

Differential diagnoses include illness anxiety disorder, panic disorder, functional neurological disorder, persistent postural-perceptual dizziness, medication adverse effects, major depressive disorder with somatic symptoms, generalized anxiety disorder, vestibular disorders, sleep disorders, and medical conditions producing pain or autonomic symptoms. Diagnostic formulation should allow more than one condition to be present. Autonomic symptoms such as palpitations, sweating, nausea, light-headedness, or hyperventilation during anxiety or depression do not by themselves establish SSD; the required symptom-related cognitive, affective, or behavioral criteria must also be present.

Respiratory complaints such as dyspnea or hyperventilation-related sensations can occur in persistent somatic-symptom presentations, but the available literature is heterogeneous and does not justify treating these symptoms as evidence of either SSD or upper-cervical disease without an appropriate differential diagnosis (19).

### 5.2 Measurement

Validated instruments can clarify, but not replace, clinical diagnosis. The PHQ-15 and SSS-8 quantify somatic symptom burden (20, 21). The SSD-12 assesses excessive cognitive, affective, and behavioral responses corresponding to the psychological criteria of SSD (22). The PHQ-9 and GAD-7 measure depressive and anxiety symptoms, respectively (23, 24), and the Hospital Anxiety and Depression Scale can be useful in medical populations (25). The Short Health Anxiety Inventory (SHAI) can be used when illness-related fear is prominent (26). Scores should be interpreted according to their intended construct: high somatic symptom severity is not itself a diagnosis of SSD.

Culture influences whether distress is expressed, recognized, and interpreted in bodily or psychological terms. Clinicians should avoid assuming that somatic presentation reflects poor insight or “psychological causation,” and should consider language, explanatory models, stigma, health-care access, and cultural norms (27).

### 5.3 Treatment principles

Treatment should validate the reality of symptoms, provide a coherent formulation, schedule regular follow-up, coordinate medical and mental-health care, and reduce repeated low-yield testing after appropriate evaluation. A stepped-care approach can incorporate psychoeducation, cognitive-behavioral therapy (CBT), mindfulness- or acceptance-based strategies, graded activity, sleep intervention, consultation-liaison psychiatry, and treatment of comorbid anxiety or depression (28, 29).

Outcomes should prioritize function, distress, quality of life, health-care use, and participation rather than complete elimination of every bodily sensation. Evidence for pharmacotherapy targeting SSD itself is limited and generally of low or very low certainty; antidepressants are more clearly indicated for a comorbid depressive or anxiety disorder than for SSD as a stand-alone diagnosis (30). Benzodiazepines should not be presented as routine SSD therapy, and prolonged use is discouraged because of tolerance, dependence, cognitive effects, falls, and withdrawal.

## 6 Potential mechanisms: what is supported and what remains hypothetical

### 6.1 Autonomic regulation — mechanistic inference

Altered heart-rate variability and autonomic regulation have been reported in anxiety and depressive disorders (31). Autonomic symptoms such as palpitations, sweating, nausea, and light-headedness can also arise during anxiety. One chronic non-specific neck-pain study reported heart-rate-variability differences, but its participants did not undergo objective C1–C2 assessment (32). The anatomical presence of sympathetic fibers near cervical vessels does not demonstrate that atlantoaxial dysfunction produces clinically significant dysautonomia. Statements linking cervical proprioception to autonomic arousal should therefore be labeled theoretical unless tested in a cohort with defined atlantoaxial disease.

### 6.2 Pain processing, hypervigilance, and predictive mechanisms — adjacent evidence

Central sensitization, symptom amplification, hypervigilance, negative affect, and predictive processing are related but distinct concepts. Central sensitization refers to altered central nociceptive responsiveness; symptom amplification is a broader clinical phenomenon; hypervigilance concerns attention to threat; and predictive-processing models address how prior expectations and sensory evidence are weighted.

General neck-pain studies support associations between distress and pain-related disability (2, 33), while one resting-state functional-connectivity study identified altered connectivity between the subgenual anterior cingulate cortex and superior temporal gyrus in patients with depression or anxiety and somatic symptoms (34). These findings may explain why people differ in the severity assigned to similar sensory input. They do not show that a minor atlantoaxial imaging feature is the source of severe dizziness or headache. An equally plausible explanation is that the imaging finding is incidental.

### 6.3 Cervical proprioception — adjacent evidence

The upper cervical region provides substantial proprioceptive input for head, eye, and postural control. Cervical sensorimotor disturbance could plausibly contribute to non-spinning disequilibrium through sensory mismatch (35). In rheumatoid arthritis, AAS was associated with impaired cervical joint-position sense, but depression had weak or non-significant relationships with proprioception (6). This finding supports a possible C1–C2 sensorimotor contribution in a specific inflammatory population; it does not establish that anxiety causes instability or that AAI commonly explains dizziness in psychiatric patients.

### 6.4 Biomechanics — a testable but unvalidated causal chain

The proposed pathway can be expressed as four transitions:

1. Anxiety or threat may increase cervical muscle activation and protective behavior.
2. Persistent activation may alter movement variability, endurance, or motor control.
3. Altered motor behavior may change segmental loading.
4. Repeated C1–C2 loading might contribute to structural disease or symptom generation.

This pathway remains hypothesis-generating. The first two transitions are biologically plausible but were not directly evaluated in the atlantoaxial literature reviewed here; the third is inferred, and the fourth has not been demonstrated longitudinally for C1–C2 instability or degeneration. Evidence relating forward-head posture or screen exposure to non-specific neck pain should not be used as proof of accelerated atlantoaxial degeneration. Future studies should measure each transition rather than cite anatomical plausibility as a completed mechanism.

### 6.5 Inflammation — highly preliminary hypothesis

Low-grade systemic inflammation has been reported in subsets of patients with depression or SSD, and inflammatory markers have been explored as correlates of symptom outcomes (36, 37). This is not equivalent to local atlantoaxial synovitis, transverse-ligament injury, bone erosion, or instability. Rheumatoid arthritis produces C1–C2 pathology through a distinct inflammatory disease process and should not be used as indirect proof that psychiatric inflammation causes AAI. At present, inflammation is a research hypothesis, not an established link between the two clinical domains.

### 6.6 Sleep, fear avoidance, and deconditioning — clinically relevant modifiers

Sleep disturbance can intensify pain, fatigue, dizziness, negative affect, and symptom monitoring. Psychosocial and sleep variables are associated with neck pain in general populations (3). Fear avoidance may reduce movement and participation, while persistent pain can further disrupt sleep and mood. These processes can maintain disability regardless of whether an initial structural lesion exists. Acute sleep-deprivation studies of muscle protein synthesis and low-back-pain fear-avoidance studies do not demonstrate impaired atlantoaxial stability and should not be cited for that purpose.

## 7 Discussion

### 7.1 Principal interpretation

The principal finding is not merely that causality remains unproven. The available literature is insufficient to quantify a specific association between objectively confirmed atlantoaxial disease and anxiety- or depression-related somatic symptom burden. Three studies measured some elements of both domains, but none was designed around the review question and none assessed SSD. The broader literature supports coexistence, symptom overlap, and shared modifiers more strongly than it supports a C1–C2-specific relationship.

Coexistence, statistical association, shared risk, symptom overlap, and causation must be separated. An individual can have both AAI and depression without either causing the other. Neck pain may worsen anxiety and depression through disability and sleep disruption, but this does not imply that psychiatric symptoms produced structural disease. Conversely, psychological distress may amplify pain or dizziness, but this does not invalidate a concordant structural lesion.

### 7.2 Confounding and alternative explanations

Important confounders include age, trauma, rheumatoid or other inflammatory disease, congenital conditions, generalized pain, migraine, sleep disturbance, medication use, physical inactivity, vestibular disease, neurological disease, and health-care-seeking behavior. These variables can influence both imaging frequency and symptom reporting. Administrative studies are especially vulnerable to confounding because depression codes can reflect comorbidity burden and service use rather than a mechanistic relationship.

Reverse causation is plausible: persistent neck pain, neurological disability, uncertainty, and repeated medical encounters may increase anxiety or depressive symptoms. Incidental imaging findings are also plausible, particularly when mild degeneration or asymmetry is found without neurological or biomechanical concordance. Both possibilities should appear explicitly in interpretation.

### 7.3 Diagnostic overshadowing in both directions

Two forms of diagnostic masking are clinically important. Psychiatric overshadowing occurs when neurological or structural symptoms are dismissed because anxiety, depression, or SSD is present. Structural overshadowing occurs when all symptoms are attributed to an imaging finding despite poor clinicoradiological concordance, thereby overlooking migraine, vestibular disease, medication effects, panic, depression, sleep disturbance, or persistent postural-perceptual dizziness. A balanced assessment should actively guard against both errors.

### 7.4 A cautious clinical pathway

A practical sequence is:

1. Screen for urgent neurological, traumatic, infectious, and vascular red flags.
2. Characterize dizziness and headache rather than assuming a cervical source. Exclude common vestibular, neurological, vascular, visual, medication-related, and psychiatric causes.
3. Define the spinal entity. Distinguish non-specific pain, stable degeneration, inflammatory disease, fixed rotatory disorder, and dynamic instability.
4. Assess clinicoradiological concordance. Interpret imaging in relation to mechanism, examination, timing, and severity; avoid repeated imaging without a new indication.
5. Assess parallel modifiers. Use validated measures for depression, anxiety, somatic symptom burden, SSD-related cognitive/behavioral features, sleep, health anxiety, and fear avoidance as clinically indicated.
6. Match referral and treatment to the dominant findings. Urgent specialty care is required for red flags; confirmed instability requires spine expertise; vestibular findings require appropriate evaluation; and clinically important affective or somatic distress warrants evidence-based mental-health or consultation-liaison care.

Integrated care is reasonable as a clinical framework, but it has not been proven superior to alternative approaches specifically in patients with atlantoaxial disease and psychiatric somatic symptoms. Claims of treatment superiority should await controlled studies.

### 7.5 Research priorities

The highest-priority study would be a prospective cohort in which participants receive standardized C1–C2 imaging and neurological assessment together with PHQ-9, GAD-7, SSS-8 or PHQ-15, SSD-12, sleep, fear-avoidance, and vestibular measures. Prespecified definitions should distinguish fixed structural abnormalities from dynamic instability and incidental variation. Longitudinal follow-up could test temporal directionality. Mechanistic substudies could evaluate cervical proprioception, autonomic regulation, pain modulation, and inflammatory markers without assuming that one domain mediates the other. Treatment trials should stratify patients by objectively confirmed pathology and report function, distress, quality of life, health-care use, and adverse events.

### 7.6 Limitations

This is a narrative rather than systematic review. Although the search was structured and transparent, screening and synthesis were conducted in a single-reviewer workflow, creating a risk of selection and interpretation bias. The terminology of AAI, AAS, rotatory fixation, upper-cervical dysfunction, somatic symptoms, SSD, and bodily distress was heterogeneous. The evidence categories required judgment, and no single formal risk-of-bias tool was applicable to all included designs. Much of the mechanistic discussion depends on indirect evidence from general neck pain, depression, SSD, sleep, or inflammation studies without C1–C2 assessment. Publication bias and selective reporting are likely, particularly in case reports and surgical series. These limitations preclude causal or prevalence estimates.

## 8 Conclusions

Anxiety- or depression-related somatic symptoms and atlantoaxial disorders may coexist and can share symptoms such as pain, headache, dizziness, fatigue, and sleep disturbance. Current evidence does not establish that either condition causes the other, does not support a bidirectional causal claim, and is insufficient to quantify a C1–C2-specific association.

The clinically useful conclusion is therefore one of diagnostic discipline. Objective atlantoaxial pathology should be defined and interpreted for clinical concordance; dizziness should undergo an appropriate differential diagnosis; and anxiety, depression, sleep disturbance, fear avoidance, somatic symptom burden, and SSD-related cognitions or behaviors should be assessed as distinct constructs. Proposed autonomic, nociceptive, proprioceptive, biomechanical, inflammatory, sleep-related, and behavioral pathways remain targets for prospective testing rather than established mechanisms.
