# Supplementary material for: Anxiety- and depression-related somatic symptoms and atlantoaxial disorders: potential mechanistic links and clinical implications
Source: Front Psychiatry. 2026 Sep 3;17:1919035. doi: 10.3389/fpsyt.2026.1919035 (PMC13581517; doi:10.3389/fpsyt.2026.1919035)
Supplement: Supplementary file 1 [file DataSheet1.pdf]

Supplementary Appendix 1. Search Strategy and Evidence-Classification Framework

S1. Review scope

This structured narrative review examined whether objectively defined atlantoaxial disorders are associated with anxiety- or depression-related somatic symptom burden. Somatic symptom disorder (SSD) was treated as a distinct diagnostic construct rather than as a synonym for having multiple physical symptoms. Non-specific neck pain, cervical dizziness, cervicogenic headache, and clinically defined upper-cervical dysfunction were treated as adjacent conditions rather than as evidence of structural C1–C2 disease. The review focused primarily on adults. Pediatric or adolescent studies were included only to clarify rotatory fixation or developmental instability, or as explicitly indirect contextual evidence when relevant adult evidence was limited.

Five bibliographic databases were searched from inception through 29 July 2026: PubMed/MEDLINE, Embase, Scopus, Web of Science Core Collection, and APA PsycInfo. No date, language, human-only, publication-type, or study-design filters were applied at the search stage. Conference records, trial registrations, case reports, reviews, and non-human studies were retained during initial retrieval so that their evidentiary role could be assessed explicitly.

Two complementary searches were conducted in every database:

1. **Direct search:** atlantoaxial/C1–C2 concepts combined with psychiatric or somatic-symptom concepts.
2. **Adjacent search:** general cervical-pain/dizziness/headache concepts combined with the same psychiatric or somatic-symptom concepts.

The adjacent search was intentionally broad. Its results were not interpreted as evidence of atlantoaxial disease; they were used to identify background clinical associations and possible mechanisms whose limited directness was explicitly acknowledged.

S2. Search yield

Table S1. Records retrieved by database and evidence stratum

Database	Direct search	Adjacent search	Total
APA PsycInfo	16	398	414
Embase	123	1,828	1,951
PubMed/MEDLINE	60	1,195	1,255
Scopus	175	3,545	3,720
Web of Science Core Collection	71	1,471	1,542
Total before deduplication	445	8,437	8,882

Across both strata, 3,657 duplicate records were removed, leaving 5,225 unique records. Of these, 315 unique records belonged to the direct-search stratum and 4,910 occurred only in the adjacent-search stratum. Assignment to the direct stratum took precedence when the same

record appeared in both searches. Three studies ultimately met the partial-direct category; none directly tested the review question, none assessed SSD, and none established causality.

S3. Database-specific strategies

The syntax below reproduces the final searches. Quotation marks, Boolean operators, field tags, and truncation symbols are shown as entered. Line breaks have been added for readability only.

S3.1 PubMed/MEDLINE

Direct search

```
(
  "Atlanto-Axial Joint"[Mesh]
  OR atlantoaxial[tiab]
  OR atlanto-axial[tiab]
  OR "atlanto axial"[tiab]
  OR "C1-C2"[tiab]
  OR "C1 C2"[tiab]
  OR "atlas axis"[tiab]
  OR "upper cervical instability"[tiab]
)
AND
(
  "Anxiety"[Mesh]
  OR "Anxiety Disorders"[Mesh]
  OR "Depression"[Mesh]
  OR "Depressive Disorder"[Mesh]
  OR "Somatic Symptom and Related Disorders"[Mesh]
  OR anxi*[tiab]
  OR depress*[tiab]
  OR "somatic symptom"[tiab]
  OR "somatic symptoms"[tiab]
  OR "somatic symptom disorder"[tiab]
  OR somatization[tiab]
  OR somatisation[tiab]
  OR psychosomatic[tiab]
  OR "functional somatic"[tiab]
  OR "persistent physical symptom"[tiab]
  OR "persistent physical symptoms"[tiab]
  OR "bodily distress"[tiab]
  OR "symptom amplification"[tiab]
  OR "health anxiety"[tiab]
  OR "illness anxiety"[tiab]
  OR "psychological distress"[tiab]
)
```

Adjacent search

```
(
  "Neck Pain"[Mesh]
  OR "neck pain"[tiab]
  OR cervicalgia[tiab]
  OR "cervical pain"[tiab]
  OR "upper cervical dysfunction"[tiab]
  OR "cervicogenic dizziness"[tiab]
  OR "cervical dizziness"[tiab]
  OR "cervical vertigo"[tiab]
  OR "cervicogenic headache"[tiab]
)
AND
(
  "Anxiety"[Mesh]
  OR "Anxiety Disorders"[Mesh]
```

```
  OR "Depression"[Mesh]
  OR "Depressive Disorder"[Mesh]
  OR "Somatic Symptom and Related Disorders"[Mesh]
  OR anxi*[tiab]
  OR depress*[tiab]
  OR "somatic symptom"[tiab]
  OR "somatic symptoms"[tiab]
  OR "somatic symptom disorder"[tiab]
  OR somatization[tiab]
  OR somatisation[tiab]
  OR psychosomatic[tiab]
  OR "functional somatic"[tiab]
  OR "persistent physical symptom"[tiab]
  OR "persistent physical symptoms"[tiab]
  OR "bodily distress"[tiab]
  OR "symptom amplification"[tiab]
  OR "health anxiety"[tiab]
  OR "illness anxiety"[tiab]
  OR "psychological distress"[tiab]
)
```

S3.2 Embase.com

Direct search

```
(
  (
    'atlantoaxial':ti,ab,kw
    OR 'atlanto-axial':ti,ab,kw
    OR 'atlanto axial':ti,ab,kw
    OR 'c1-c2':ti,ab,kw
    OR 'c1 c2':ti,ab,kw
    OR 'atlas axis':ti,ab,kw
    OR 'upper cervical instability':ti,ab,kw
  )
  AND
  (
    anxi*:ti,ab,kw
    OR depress*:ti,ab,kw
    OR 'somatic symptom':ti,ab,kw
    OR 'somatic symptoms':ti,ab,kw
    OR 'somatic symptom disorder':ti,ab,kw
    OR somatization:ti,ab,kw
    OR somatisation:ti,ab,kw
    OR psychosomatic:ti,ab,kw
    OR 'functional somatic':ti,ab,kw
    OR 'persistent physical symptom':ti,ab,kw
    OR 'persistent physical symptoms':ti,ab,kw
    OR 'bodily distress':ti,ab,kw
    OR 'symptom amplification':ti,ab,kw
    OR 'health anxiety':ti,ab,kw
    OR 'illness anxiety':ti,ab,kw
    OR 'psychological distress':ti,ab,kw
  )
)
```

Adjacent search

```
(
  'neck pain':ti,ab,kw
  OR cervicalgia:ti,ab,kw
  OR 'cervical pain':ti,ab,kw
  OR 'upper cervical dysfunction':ti,ab,kw
  OR 'cervicogenic dizziness':ti,ab,kw
  OR 'cervical dizziness':ti,ab,kw
  OR 'cervical vertigo':ti,ab,kw
  OR 'cervicogenic headache':ti,ab,kw
```

```
)
AND
(
  anxi*:ti,ab,kw
  OR depress*:ti,ab,kw
  OR 'somatic symptom':ti,ab,kw
  OR 'somatic symptoms':ti,ab,kw
  OR 'somatic symptom disorder':ti,ab,kw
  OR somatization:ti,ab,kw
  OR somatisation:ti,ab,kw
  OR psychosomatic:ti,ab,kw
  OR 'functional somatic':ti,ab,kw
  OR 'persistent physical symptom':ti,ab,kw
  OR 'persistent physical symptoms':ti,ab,kw
  OR 'bodily distress':ti,ab,kw
  OR 'symptom amplification':ti,ab,kw
  OR 'health anxiety':ti,ab,kw
  OR 'illness anxiety':ti,ab,kw
  OR 'psychological distress':ti,ab,kw
)
```

S3.3 Scopus

Direct search

```
TITLE-ABS-KEY(
  "atlantoaxial" OR "atlanto-axial" OR "atlanto axial"
  OR "C1-C2" OR "C1 C2" OR "atlas axis"
  OR "upper cervical instability"
)
AND
TITLE-ABS-KEY(
  anxi* OR depress* OR "somatic symptom" OR "somatic symptoms"
  OR "somatic symptom disorder" OR somatization OR somatisation
  OR psychosomatic OR "functional somatic"
  OR "persistent physical symptom" OR "persistent physical symptoms"
  OR "bodily distress" OR "symptom amplification"
  OR "health anxiety" OR "illness anxiety"
  OR "psychological distress"
)
```

Adjacent search

```
TITLE-ABS-KEY(
  "neck pain" OR cervicalgia OR "cervical pain"
  OR "upper cervical dysfunction" OR "cervicogenic dizziness"
  OR "cervical dizziness" OR "cervical vertigo"
  OR "cervicogenic headache"
)
AND
TITLE-ABS-KEY(
  anxi* OR depress* OR "somatic symptom" OR "somatic symptoms"
  OR "somatic symptom disorder" OR somatization OR somatisation
  OR psychosomatic OR "functional somatic"
  OR "persistent physical symptom" OR "persistent physical symptoms"
  OR "bodily distress" OR "symptom amplification"
  OR "health anxiety" OR "illness anxiety"
  OR "psychological distress"
)
```

S3.4 Web of Science Core Collection

Direct search

```
TS=(
  "atlantoaxial" OR "atlanto-axial" OR "atlanto axial"
  OR "C1-C2" OR "C1 C2" OR "atlas axis"
```

```
  OR "upper cervical instability"
)
AND
TS=(
  anxi* OR depress* OR "somatic symptom" OR "somatic symptoms"
  OR "somatic symptom disorder" OR somatization OR somatisation
  OR psychosomatic OR "functional somatic"
  OR "persistent physical symptom" OR "persistent physical symptoms"
  OR "bodily distress" OR "symptom amplification"
  OR "health anxiety" OR "illness anxiety"
  OR "psychological distress"
)
```

Adjacent search

```
TS=(
  "neck pain" OR cervicalgia OR "cervical pain"
  OR "upper cervical dysfunction" OR "cervicogenic dizziness"
  OR "cervical dizziness" OR "cervical vertigo"
  OR "cervicogenic headache"
)
AND
TS=(
  anxi* OR depress* OR "somatic symptom" OR "somatic symptoms"
  OR "somatic symptom disorder" OR somatization OR somatisation
  OR psychosomatic OR "functional somatic"
  OR "persistent physical symptom" OR "persistent physical symptoms"
  OR "bodily distress" OR "symptom amplification"
  OR "health anxiety" OR "illness anxiety"
  OR "psychological distress"
)
```

S3.5 APA PsycInfo via EBSCOhost

The following Boolean strings were entered without an explicit field limiter in the EBSCOhost APA PsycInfo interface. No post-search filters were applied.

Direct search

```
(
  "atlantoaxial" OR "atlanto-axial" OR "atlanto axial"
  OR "C1-C2" OR "C1 C2" OR "atlas axis"
  OR "upper cervical instability"
)
AND
(
  anxi* OR depress* OR "somatic symptom" OR "somatic symptoms"
  OR "somatic symptom disorder" OR somatization OR somatisation
  OR psychosomatic OR "functional somatic"
  OR "persistent physical symptom" OR "persistent physical symptoms"
  OR "bodily distress" OR "symptom amplification"
  OR "health anxiety" OR "illness anxiety"
  OR "psychological distress"
)
```

Adjacent search

```
(
  "neck pain" OR cervicalgia OR "cervical pain"
  OR "upper cervical dysfunction" OR "cervicogenic dizziness"
  OR "cervical dizziness" OR "cervical vertigo"
  OR "cervicogenic headache"
)
AND
(
  anxi* OR depress* OR "somatic symptom" OR "somatic symptoms"
```

```
  OR "somatic symptom disorder" OR somatization OR somatisation
  OR psychosomatic OR "functional somatic"
  OR "persistent physical symptom" OR "persistent physical symptoms"
  OR "bodily distress" OR "symptom amplification"
  OR "health anxiety" OR "illness anxiety"
  OR "psychological distress"
)
```

S4. Eligibility, selection, and evidence classification

Table S2. Evidence categories used in the narrative synthesis

Category	Operational definition	Typical eligible evidence	Interpretation
Direct or partial-direct evidence	Psychiatric or somatic-symptom data and an upper-cervical assessment were available in the same participants. Evidence was classified as direct when objectively defined C1–C2 pathology and a targeted psychiatric or somatic-symptom measure were analyzed in relation to one another. Evidence was classified as partial-direct when that relationship was not directly tested, or when a spinal or psychiatric variable relied on administrative coding or a non-targeted assessment	Imaging-confirmed C1–C2 cohort with targeted psychiatric or SSD measures (direct); imaging or administrative cohort with non-targeted or administratively coded psychiatric data (partial-direct)	Closest to the review question; directness and causal inference remain limited by study design, measurement quality, and control of confounding
Adjacent evidence	Mental-health variables assessed in non-specific neck pain, cervical radiculopathy, whiplash, cervicogenic headache, or cervical-dizziness populations without a specific C1–C2 diagnosis	Observational cervical-pain cohorts and cervical-dizziness reviews	Supports contextual association only; cannot establish atlantoaxial disease
Mechanistic inference	Autonomic, inflammatory, interoceptive, nociceptive, proprioceptive, sleep, or behavioral findings without atlantoaxial assessment	Mechanistic studies in depression, SSD, sleep disturbance, or general neck pain	Biological plausibility; no C1–C2-specific clinical validation
Hypothesis-generating evidence	Small uncontrolled observation or biologically plausible pathway not validated in a comparative human study	Case reports, case series, anatomical arguments	Generates testable hypotheses; not evidence of frequency, diagnostic accuracy, efficacy, or causality

Records were excluded from the evidentiary synthesis when the search terms were used in unrelated contexts; atlantoaxial disease and psychiatric symptoms were listed as separate comorbidities without an analyzed relationship; a case report was being used to imply frequency, diagnostic accuracy, or treatment efficacy; or evidence from general neck pain

was presented as proof of structural C1–C2 disease. Foundational anatomy, diagnostic guidance, consensus statements, measurement studies, and systematic reviews were retained when they supported definition, differential diagnosis, or clinical context.

Titles and abstracts were screened in a single-reviewer workflow using these predefined criteria. Potentially relevant records from the direct-search stratum and key adjacent studies underwent full-record assessment. Because screening was undertaken by one reviewer, no inter-reviewer disagreement-resolution procedure was applicable; this creates a risk of selection and interpretation bias. Study design, sample, spinal diagnosis and assessment, psychiatric or somatic-symptom measure, principal result, directness, confounding, and major limitations were extracted for the evidence table. Citation lists of key reviews and consensus statements were checked for foundational sources.

A single formal risk-of-bias instrument was not applied because the evidence base contained heterogeneous diagnostic reviews, consensus statements, cross-sectional cohorts, administrative studies, mechanistic studies, and case reports. Instead, appraisal considered design, diagnostic validity, directness to objectively confirmed C1–C2 disease, psychiatric measurement quality, confounding, and precision. Evidence selection and citation were based on clinical directness, diagnostic validity, methodological relevance, and evidentiary role rather than recency. Recent publications were not preferred solely because of year; foundational diagnostic, consensus, measurement, and mechanistic sources were retained when authoritative. This judgment-based approach and the single-reviewer workflow are acknowledged limitations.

S5. Deduplication and interpretation safeguards

Records were merged across databases and deduplicated using DOI, PMID, normalized title, year, and other available bibliographic fields. Database provenance was retained. Records found by both the direct and adjacent searches were assigned to the direct-search stratum to avoid double counting.

The final synthesis did not use raw record counts as evidence of an association. The counts describe retrieval only. Direct-search false positives were common, including papers in which atlantoaxial disease and depression were unrelated comorbidities, “C1–C2” denoted a coordinate or classification axis, or a psychiatric term appeared only in background text. The conclusion was therefore based on clinical directness and study design rather than search frequency.
